# Dietary Management of Atherogenic Dyslipidemia

**DOI:** 10.1007/s11883-025-01335-6

**Published:** 2025-09-15

**Authors:** Shieon Kim, Min-Jeong Shin, Ronald M. Krauss

**Affiliations:** 1 Interdisciplinary Program in Precision Public Health, Graduate School of Korea University, Seoul, Korea; 2 School of Biosystems and Biomedical Sciences, College of Health Science, Korea University, Seoul, Korea; 3 Departments of Pediatrics and Medicine, University of California, San Francisco, CA USA

**Keywords:** Atherogenic dyslipidemia, Small dense LDL (sdLDL), Dietary intervention, Carbohydrate restriction

## Abstract

**Purpose of Review:**

This paper reviews the effects of major macronutrients and specific dietary interventions on atherogenic dyslipidemia, a common trait characterized by increased concentrations of triglyceride-rich and small, dense LDL particles, and reduced HDL-cholesterol.

**Recent Findings:**

Studies have shown that reducing carbohydrate intake is the most effective dietary approach for managing atherogenic dyslipidemia, particularly in individuals with excess adiposity and/or metabolic syndrome. Plant protein sources can also be beneficial, possibly due to their content of phytochemicals. Whereas dietary guidelines emphasize limiting intake of saturated fat for reducing cardiovascular risk by lowering concentrations of LDL cholesterol, this has not been shown to have an impact on atherogenic dyslipidemia.

**Summary:**

Attenuation or reversal of atherogenic dyslipidemia can be achieved by adopting a dietary pattern that emphasizes moderating carbohydrate intake, in particular processed grains and added sugars, rather than by focusing primarily on limiting saturated fat and its effects on LDL-cholesterol.

## Introduction

Atherogenic dyslipidemia is a highly prevalent metabolic trait that is strongly associated with risk for atherosclerotic cardiovascular disease (ASCVD), the major cause of mortality in the U.S. and globally [1]. It is characterized by interrelated lipid and lipoprotein abnormalities including elevated serum triglycerides (TG) and TG-rich very low-density lipoproteins (VLDLs), decreased high-density lipoprotein cholesterol (HDL-C) [2], and increased concentrations of small, dense low-density lipoprotein (sdLDL) particles [3]. Moreover, it is a major component of metabolic syndrome, and as such is associated with excess abdominal adiposity and insulin resistance [4] as well as chronic inflammation [5]. The relationship of atherogenic dyslipidemia to excess adiposity is further highlighted by our finding that LDL subclass B, which designates a lipoprotein phenotype characterized by a predominance of sdLDL, is associated with impaired fatty acid oxidation [6, 7]. This may therefore point to a fundamental defect contributing to both increased adiposity and atherogenic dyslipidemia [8, 9] and a greater dependence on carbohydrates vs. fats as an energy source in individuals with this metabolic trait.

Dietary management of atherogenic dyslipidemia is a cornerstone for reducing its impact on ASCVD. While reduction of excess adiposity is clearly a priority, its benefit has been extensively documented [10, 11] and will not be reviewed here. Rather, we focus on the effects of the major macronutrients, independent of their potential impact on body weight, with particular emphasis on dietary measures for reducing concentrations of atherogenic TG-rich and small dense lipoprotein particles.

## Features and Determinants of Atherogenic Dyslipidemia

**Fig. 1:**
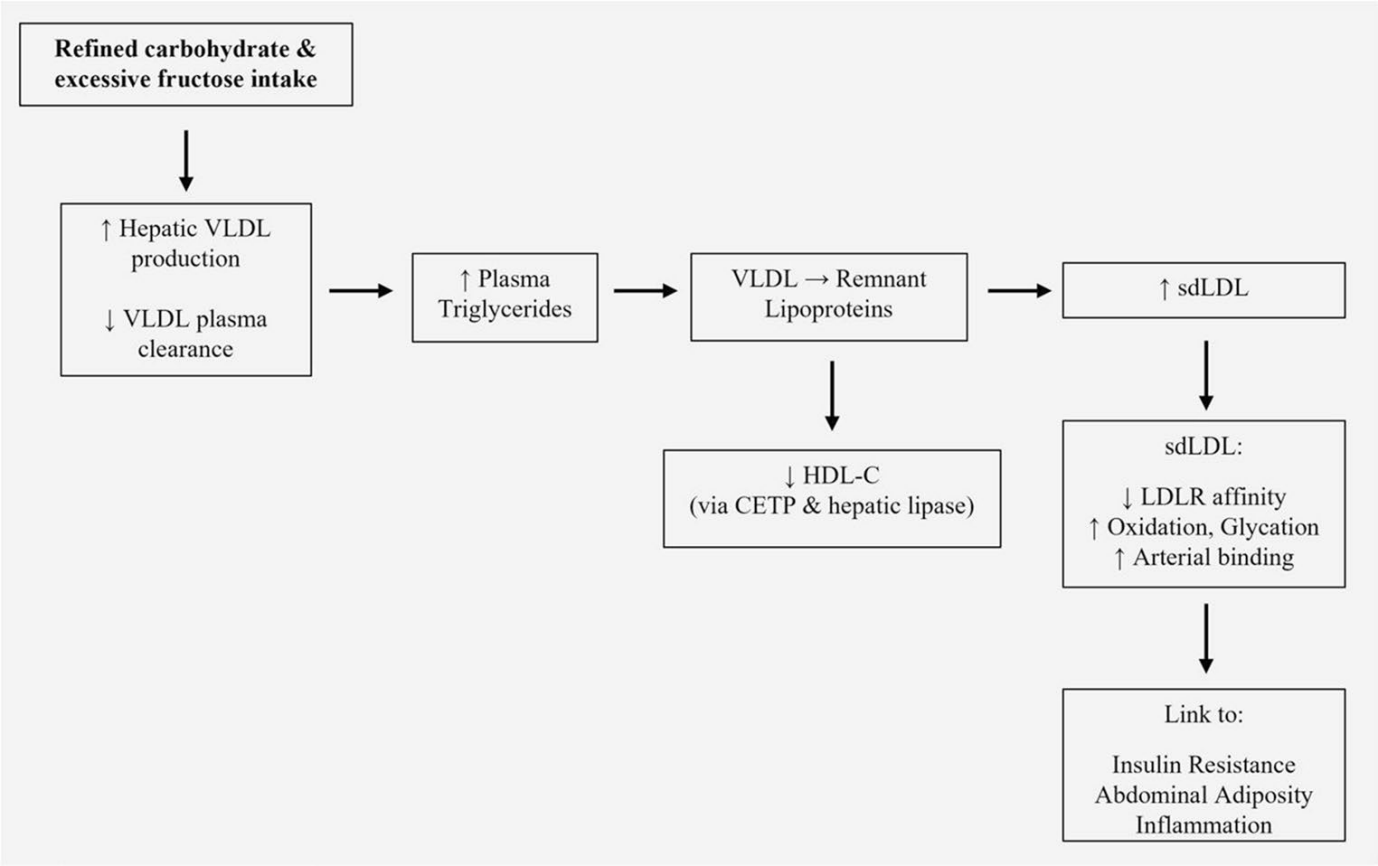
Pathophysiology of atherogenic dyslipidemia. This figure summarizes the core mechanisms of atherogenic dyslipidemia, including increased VLDL and triglycerides, reduced HDL-C, and accumulation of sdLDL, and its relation to insulin resistance, visceral adiposity, and inflammation

Atherogenic dyslipidemia has a high prevalence in individuals with insulin resistance and obesity. According to data from the National Health and Nutrition Examination Survey (NHANES), the percentage of individuals receiving treatment for dyslipidemia increased significantly between 2001 and 2020 [12]. In the U.S., approximately 35–50% of people with type 2 diabetes have dyslipidemia, typically with two or more lipid abnormalities [13]. The TG elevation in atherogenic dyslipidemia is related to both increased hepatic production and reduced plasma clearance of VLDL particles [14, 15]. Hepatic insulin resistance plays a key role by stimulating de novo lipogenesis and failing to suppress the secretion of ApoB-containing lipoproteins [16]. In addition, reduced activity of lipoprotein lipase delays the plasma clearance of VLDL particles [16]. As a result, an excess of triglyceride-rich VLDL contributes to the formation of remnant lipoproteins, which are strongly associated with elevated ASCVD risk [14, 17]. These remnant particles can be further remodeled by hepatic lipase and cholesteryl ester transfer protein (CETP), resulting in the production of sdLDL particles and a parallel reduction in HDL-C and large HDL subfractions (Fig. 1) [18]. This lipoprotein remodeling cascade becomes more pronounced in the context of hypertriglyceridemia, frequently driven by high-carbohydrate diets that promote hepatic VLDL synthesis and facilitate sdLDL generation.

Despite the well-established association of low HDL-C to risk of ASCVD [19], assessment of its independent effect on risk is complicated by its relationship to the other constituents of atherogenic dyslipidemia. Moreover, the potential benefits of increasing HDL on ASCVD risk have been called into question by studies employing Mendelian randomization [20] as well as clinical trials that have failed to show a benefit of HDL-raising pharmacologic therapies [21, 22], and the recent evidence that very high HDL-C concentrations are associated with increased ASCVD mortality [19, 23].

As is the case for HDL-C, the interrelationships of sdLDL with the other components of atherogenic dyslipidemia have made it challenging to determine its independent effects on ASCVD risk. Importantly, however, concentrations of relatively cholesterol-depleted sdLDL particles have been associated with incidence of ASCVD independent of total LDL-cholesterol (LDL-C) and other conventional risk factors. In contrast, large buoyant LDL (lbLDL) have been shown to have a much weaker or negligible relationship to ASCVD risk [24]. Multiple properties distinguish sdLDL from lbLDL in terms of atherogenic potential. These include longer plasma residence time due to decreased LDL receptor affinity, greater binding to arterial proteoglycans, and enhanced susceptibility to oxidative modification and glycation. sdLDL particles also carry a higher content of the proinflammatory protein apolipoprotein C-III [24]. These findings underscore the importance of sdLDL as a key marker of ASCVD risk. They also challenge the conventional reliance on total LDL-C for risk estimation, as well as the notion that all LDL and apoB-containing particles are equally atherogenic, as discussed elsewhere [24]. For these reasons, therapies aimed at reducing sdLDL present a strong opportunity for reducing ASCVD risk.

## Dietary Effects on Atherogenic Dyslipidemia

Dietary composition has an important influence on lipoprotein metabolism associated with atherogenic dyslipidemia. sdLDL particles are generated through a stepwise remodeling process of TG-rich VLDL particles [18]. Insulin resistance in the liver induces overproduction of VLDL, and the actions of delayed lipoprotein lipase and hepatic lipase promote the gradual conversion of VLDL into smaller, denser LDL particles [18]. To modulate the production of these sdLDL, it is important to understand the dietary factors that influence the lipoprotein remodeling pathway. Saturated fatty acids (SFAs) intake causes an increase in LDL-C but generally has no significant effect on sdLDL [25]. On the other hand, refined carbohydrate [25] and excessive fructose intake promote VLDL overproduction [26], and conversely, dietary fiber is known to reduce TG concentrations and inhibit sdLDL production [27]. In addition, factors such as the type of fatty acid [25] and animal vs. plant protein [28] may also influence sdLDL formation. In this section, we specifically examine the differential effects of fat, carbohydrate, protein, and plant components on sdLDL formation and lipoprotein metabolism.

### Fats

Dietary guidelines for reducing LDL concentrations and CVD risk have consistently included limiting intake of SFAs, principally based on the many studies that have shown that higher SFA intake raises LDL-C concentrations. However, it has been shown that in general dietary SFAs primarily increase larger cholesterol- rich LDL particles, with little effect on sdLDL (Table 1) [25, 29–31]. This can be accounted for by the reduced LDL receptor affinity of sdLDL [24] since LDL receptor suppression is the main mechanism by which SFAs raise LDL concentrations [32]. Consequently, the effects of dietary SFA limitation on ASCVD risk may be overestimated by focusing solely on LDL-C response.

**Table 1:** Effects of macronutrients on atherogenic lipoproteins. Summary of the evidence for effects of major macronutrients on atherogenic lipoprotein profiles, including TG, sdLDL, lbLDL, HDL-C, and total LDL-C

Lipids/Lipoprotein	TG	sdLDL	lbLDL	HDL-C	Total LDL-C
Nutrient					
SFA	↔	↔	↑	↔/↑	↑
PUFA	↓	↓ (slight)	↓	↑	↓
Added sugar & refined carbohydrates	↑	↑	↔/↓	↓	↔
Protein (animal vs. plant)	↔	↔	↑	↔	↓

Moreover, individual SFAs differ in their effects on LDL metabolism and considering them as a single dietary component also overlooks the differing impact on ASCVD risk of the foods in which they are consumed [33]. This is illustrated most notably in the case of regular-fat vs. low-fat dairy foods. Despite their differing SFA content, current evidence does not support a difference in their impact on ASCVD, as reviewed recently by an expert panel [34]. In addition, higher intake of fermented dairy products such as yogurt, as well as tissue and blood levels of C15:0 and C17:0 SFAs, which are biomarkers of dairy intake, have been associated with beneficial cardiometabolic effects, including reduced risk of type 2 diabetes [33, 35, 36]. The importance of the complex matrix of various dairy foods is supported by evidence that, at comparable intakes of SFAs, LDL-C concentrations are lower with consumption of cheese compared to butter [25, 37, 38]. This matrix includes differing contents of proteins, minerals, phospholipids, probiotics, bioactive peptides, and vitamins such as K2 [33]. Finally, we have shown that the well-documented blood pressure lowering effect of the DASH eating plan, attributed in part to dairy products [39], is preserved even with high intake of full-fat vs. low- and nonfat dairy foods. This dietary pattern also results in reduced concentrations of TG and VLDL, without increasing LDL-C.

Although the impact of SFAs may vary depending on the context, replacing them with unsaturated fats is still widely supported to reduce ASCVD risk. Substituting approximately 5–10% of total energy intake from SFAs with omega-6 polyunsaturated (PUFA) has been shown to improve lipid profiles and reduce cardiovascular risk [25, 40]. Monounsaturated fatty acids (MUFA) may also improve lipid profiles and reduce the risk of ASCVD [25], a finding further supported by recent lipidomic analyses from the DIVAS trial and EPIC-Potsdam cohort, where replacing SFAs with unsaturated fats led to favorable changes across multiple lipid parameters and a substantially lower risk of cardiometabolic diseases [41]. As discussed below, replacement of SFAs by refined carbohydrates can exacerbate atherogenic dyslipidemia by increasing TG and sdLDL concentrations (Table 2) [25].

**Table 2:** Effects of macronutrient replacement. Summary of the effects of macronutrient replacement on lipid profiles, particularly focusing on changes in TG, LDL-C, and sdLDL

Replaced Nutrient	Replaced By	Effect
SFA	PUFA/MUFA	↓ LDL-C *(large LDL)*
SFA	Refined Carbs	↑ TG, ↑ sdLDL
Carbohydrates	Protein	↓ TG, ↓ sdLDL

Lastly, it has been reported that dietary supplementation with the omega-3 fatty acid eicosatetraenoic acid (EPA) in individuals with metabolic syndrome significantly reduced both the concentrations and proportion of sdLDL particles [42]. These effects of EPA may contribute to the cardiovascular benefits associated with adherence to a Mediterranean diet [43].

### Carbohydrates

High intakes of processed grains and added sugars can induce or amplify the features of atherogenic dyslipidemia (Table 1) [29]. Excessive fructose intake (> 25% of total energy) has been found to hinder plasma TG clearance and enhance *de novo* lipogenesis, resulting in elevated TG, sdLDL, apo B, and non-HDL cholesterol [26]. These effects are especially pronounced in individuals with metabolic syndrome, in whom consuming substantially greater amounts of fructose than glucose has been reported to double sdLDL concentrations [26]. Moreover, levels of circulating even-chain SFAs such as palmitic acid, which can have adverse inflammatory effects on ASCVD risk, are impacted to a greater extent by stimulation of *de novo* lipogenesis on a high carbohydrate diet than by conventional dietary intakes of SFAs [33].

We have shown in a randomized clinical trial that in overweight and obese individuals with a high prevalence of atherogenic dyslipidemia, reducing dietary carbohydrate intake from 54 to 39% to 26% of total calories resulted in progressive reductions in TG and sdLDL concentrations. These effects occurred without any change in body weight and were independent of high vs. low SFA intake [29]. While most studies on carbohydrate restriction and sdLDL have focused on individuals with overweight or obesity, evidence in normal-weight populations is limited, highlighting an important area for future investigation.

Further limitation of total carbohydrate intake to a very low level that induces nutritional ketosis was also found to yield low concentrations of sdLDL in individuals with metabolic syndrome [44] and patients with diabetes [45]. A concern with consumption of such diets is that they can induce marked increases in plasma LDL-C to concentrations that may be as high as those seen in familial hypercholesterolemia, but to date this phenomenon has been observed primarily in lean individuals with relatively low TG and high HDL-C concentrations [46]. In addition, the substantial restriction of carbohydrate-rich plant foods may result in insufficient dietary fiber intake, which could negatively affect gut microbiota composition and long-term cardiometabolic health [47].

The adverse effects of dietary carbohydrates on atherogenic dyslipidemia can be mitigated by selection of minimally processed, fiber-dense food sources, such as whole grains and legumes. Soluble dietary fibers such as psyllium can lower LDL concentrations through multiple mechanisms including gut microbiome modulation and production of beneficial fermentation products [48]. It has been reported that psyllium supplements reduced sdLDL particles in obese adolescents, with a randomized controlled trial showing an approximate 1 mg/dL reduction after 7 weeks of 10 g/day intake [27].

### Proteins

Replacing dietary carbohydrates with protein can reduce features of atherogenic dyslipidemia, including TG, sdLDL, apoB, and total cholesterol: HDL-C ratio, effects that may vary depending on the protein source and saturated fat intake (Table 2) [49].

The impact of differing dietary protein sources on atherogenic dyslipidemia was assessed in a controlled randomized dietary clinical trial. This study showed that, compared to non-meat protein sources, comparable intakes of both red and white meat protein within diets containing the same amount of SFA resulted in similarly higher LDL-C and apoB concentrations. This was primarily due to increases in large LDL particles, whereas small and medium LDL, TG, and total/HDL cholesterol were unaffected by protein source (Table 1) [28]. The basis for these differing protein effects has not been explained, though, as noted below, the presence of cholesterol-lowering phytochemicals in plant foods may play a role.

### Plant Foods and Phytochemicals

Many fruits and vegetables may exert beneficial effects on ASCVD risk through antioxidant, anti-inflammatory, and endothelial-protective properties of their polyphenolic compounds. For example, flavonoids such as quercetin (found in onions and apples) [50] and anthocyanins (found in berries and red cabbage) [51] have been shown to reduce oxidative stress and improve vascular function. There is also evidence that certain plant foods and phytochemicals can reduce levels of the sdLDL component of atherogenic dyslipidemia. Specifically, reductions in sdLDL have been reported with consumption of avocados [52, 53], strawberries [54], and pistachios [55]. Additionally, a combination diet including plant sterols resulted in significant cholesterol reductions in all LDL subfractions, including sdLDL [56, 57]. In a randomized controlled trial involving children with hypercholesterolemia, daily consumption of 2 g of plant sterols in yogurt over a period of 6 to 12 months led to a significant reduction in sdLDL-C concentrations [57]. Among studies of other phytochemicals, dietary supplementation with Bergavit^®^ (150 mg/day of flavonoids: 16% neoeriocitrin, 47% neohesperidin, and 37% naringin) for 6 months in adults with moderate hypercholesterolemia was found to significantly reduce triglycerides and sdLDL concentrations [58]. While the mechanisms for these effects have not been elucidated, an animal study has reported that the polyphenolic fraction of bergamot fruit can influence lipid transfer pathways that are involved in sdLDL formation [59].

## Summary

Increased TG and sdLDL, along with decreased HDL-C, are hallmarks of atherogenic dyslipidemia, a major component of metabolic syndrome. Properties of sdLDL including reduced LDL receptor affinity, enhanced vulnerability to oxidation and glycation, and greater binding to arterial proteoglycans, play a major role in determining the ASVCD impact of this metabolic phenotype.

While dietary guideline has emphasized limitation of SFAs for LDL-C lowering, this is due primarily to reduction of larger LDL particles rather than sdLDL, and the health effects of SFAs are influenced by their food matrix and overall nutrient composition. On the other hand, limiting intakes of refined carbohydrates and added sugars reduce TG-rich lipoproteins and sdLDL, particularly in individuals with metabolic syndrome, and replacing carbohydrates, especially with plant-based proteins, can improve atherogenic lipid profiles. Certain plant foods and phytochemicals have also been shown to have the potential to reduce sdLDL concentrations.

Overall, we highlight the complex interactions between dietary macronutrients and lipid metabolism in atherogenic dyslipidemia, emphasizing the importance of both the quantity and type of carbohydrates, though macronutrient-based analysis may not be sufficient to reflect their effects at the whole food level.

## Conclusion and Future Directions

A key dietary approach for management of atherogenic dyslipidemia, beyond efforts to reduce excess adiposity, is limitation of carbohydrate intake, most importantly added sugars and processed grains. The resulting reduction of TG can lead to lower plasma concentrations of VLDL remnant and sdLDL particles which are the major features of atherogenic dyslipidemia linked to increased risk of ASCVD, and these benefits can be achieved even in the absence of weight loss. Lower LDL-C concentrations can result from substitution of unsaturated fatty acids for SFAs, and from consumption of plant vs. meat sources of dietary protein. However, these reductions are primarily due to effects on larger LDL particles, which have a much weaker association with ASCVD risk than sdLDL and are not an intrinsic component of atherogenic dyslipidemia. Thus, beyond focusing solely on achieving LDL-C reduction, a dietary approach targeting atherogenic dyslipidemia should emphasize modification of carbohydrate quantity and quality. Future studies will be required to assess the long-term effects of such a dietary approach on metabolic and cardiovascular health in diverse populations, and to investigate the role of genetic and other factors that can contribute to individual variability in dietary response [60].

## Data Availability

No datasets were generated or analysed during the current study.
